# The Effects of Methylphenidate on Cognitive Control in Active Methamphetamine Dependence Using Functional Magnetic Resonance Imaging

**DOI:** 10.3389/fpsyt.2014.00020

**Published:** 2014-03-06

**Authors:** Reem K. Jan, Joanne C. Lin, Donald G. McLaren, Ian J. Kirk, Rob R. Kydd, Bruce R. Russell

**Affiliations:** ^1^School of Pharmacy, University of Auckland, Auckland, New Zealand; ^2^Centre for Brain Research, University of Auckland, Auckland, New Zealand; ^3^Department of Radiology, Athinoula A. Martinos Center for Biomedical Imaging, Massachusetts General Hospital, Boston, MA, USA; ^4^Department of Neurology, Massachusetts General Hospital, Boston, MA, USA; ^5^Harvard Medical School, Boston, MA, USA; ^6^Department of Psychology, University of Auckland, Auckland, New Zealand; ^7^Department of Psychological Medicine, University of Auckland, Auckland, New Zealand

**Keywords:** cognitive control, BOLD, drug dependence, fMRI, methamphetamine, methylphenidate, Stroop

## Abstract

Methamphetamine (MA) dependence is associated with cognitive deficits. Methylphenidate (MPH) has been shown to improve inhibitory control in healthy and cocaine-dependent subjects. This study aimed to understand the neurophysiological effects before and after acute MPH administration in active MA-dependent and control subjects. Fifteen MA-dependent and 18 control subjects aged 18–46 years were scanned using functional magnetic resonance imaging before and after either a single oral dose of MPH (18 mg) or placebo while performing a color-word Stroop task. Baseline accuracy was lower (*p* = 0.026) and response time (RT) was longer (*p* < 0.0001) for the incongruent compared to congruent condition, demonstrating the task probed cognitive control. Increased activation of the dorsolateral prefrontal cortex (DLPFC) and parietal cortex during the incongruent and Stroop effect conditions, respectively was observed in MA-dependent compared to control subjects (*p* < 0.05), suggesting the need to recruit neural resources within these regions for conflict resolution. Post- compared to pre-MPH treatment, increased RT and DLPFC activation for the Stroop effect were observed in MA-dependent subjects (*p* < 0.05). In comparison to MPH-treated controls and placebo-treated MA-dependent subjects, MPH-treated MA-dependent subjects showed decreased activation of parietal and occipital regions during the incongruent and Stroop effect conditions (*p* < 0.05). These findings suggest that in MA-dependent subjects, MPH facilitated increased recruitment of the DLPFC for Stroop conflict resolution, and a decreased need for recruitment of neural resources in parietal and occipital regions compared to the other groups, while maintaining a comparable level of task performance to that achieved pre-drug administration. Due to the small sample size, the results from this study are preliminary; however, they inform us about the effects of MPH on the neural correlates of cognitive control in active MA-dependent subjects.

## Introduction

Methamphetamine (MA) dependence is a global public health problem, with a consumer market of between 15 and 16 million people in 2007 (1). Neuroimaging studies have shown chronic MA use in humans to be associated with cognitive deficits (2–8), decreased gray matter density or volume (9–12), increased white matter hyperintensities (13), and decreased white matter integrity (14), which are thought to reflect the toxic effects of MA on monoaminergic neurons (15–21).

Cognitive control is a broad psychological construct that refers to the ability to: select contextually relevant information (e.g., attentional selection of task-relevant stimulus information), set goals, maintain goal-directed behaviors, monitor performance, and optimally adjust behavior based on the contextually relevant information (22). As such, cognitive control is often viewed as a top-down component of cognition (22). Impairment of cognitive control, or top-down processing, is thought to be essential in the transition from casual and voluntary drug use to drug dependence in humans (5, 23–25). Functions of cognitive control, while separable, are integrally related and include: task switching, reward-based learning, performance monitoring, and conflict resolution (22). The latter includes response inhibition, and more specifically the ability to inhibit a prepotent automated response in favor of a less familiar task-relevant response (22, 26). A common measure of this type of response inhibition is the color-word Stroop task (26), which is the focus of the current study.

The color-word Stroop task consists of two conditions of interest: the congruent condition, where the word color matches the word meaning (e.g., the word “
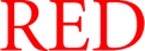
” displayed in red font color) and incongruent condition, where the word color is incompatible with the word meaning (e.g., the word “
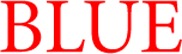
” displayed in red font color). In both instances, the correct response is “red.” Therefore, the incongruent condition requires the subject to respond to a task-relevant but less familiar dimension of a stimulus (e.g., the font color of a color-word) while inhibiting the conflicting task-irrelevant but prepotent dimension of that stimulus (e.g., reading the word) (26, 27). The difference in mean response time (RT) between the incongruent and congruent conditions results in the Stroop effect RT, also known as the Stroop interference score. A smaller interference score reflects better response inhibition and consequently better cognitive control (26, 27).

Behavioral studies of active MA-dependent subjects have reported impairments in memory, manipulation of information, and response inhibition or ability to inhibit/suppress a habitual prepotent response (e.g., word reading) in favor of an unusual but task-relevant response (e.g., color-naming) during performance of the color-word Stroop task (28, 29). Additionally, similar findings have been found in MA-dependent subjects after short-term abstinence (30, 31). These studies show that the color-word Stroop task is sensitive in detecting impairments in response inhibition in MA-dependent subjects. While neuropsychological tests can be used to study behavioral performance during cognitive control, they do not provide insight about the underlying neural mechanisms. Pinpointing neural correlates of cognitive control processes is important for studying mechanisms behind behavioral deficits induced by MA use and may be used to define appropriate interventions for treating MA dependence.

The Stroop task has been combined with neuroimaging to study regional brain activations related to cognitive control and the Stroop effect. Functional magnetic resonance imaging (fMRI) studies of cognitive control in healthy subjects have implicated the prefrontal cortex (PFC) (22, 32, 33), particularly the dorsolateral prefrontal cortex (DLPFC) (34–39), the anterior cingulate cortex (ACC) (34, 36, 37, 40–45), the parietal cortex (46), and other brain regions including inferior frontal regions and the insula (47).

Neuroimaging studies of cognitive control in MA dependence are few and have only been conducted in abstinent subjects. Abstinent MA-dependent subjects displayed behavioral deficits in cognitive control and corresponding deficits in fMRI activation of frontal and cingulate brain regions essential for cognitive control during performance of the Go/No-go task (3) and the color-word Stroop task (5). However, another study found no group differences in Stroop task performance between abstinent MA-dependent and control subjects and although they reported significant activation of the ACC corresponding with the Stroop conflict effect (incongruent–congruent), this was across all subjects with no significant group differences (8).

To date, the treatment of MA dependence has consisted primarily of psychosocial interventions including cognitive-behavioral therapy and contingency management (48), which are not often used systematically (49) and can be costly. Although several pharmacological agents have been investigated for effectiveness in improving outcomes related to the treatment of MA dependence, such as decreasing MA craving or use, most agents failed to show effectiveness (48, 50, 51). However, certain drugs have shown promise or were associated with limited reductions in MA or amphetamine use, including the dopaminergic agents, modafinil (52, 53), bupropion (54, 55), dexamphetamine (56), and methylphenidate (MPH) (51, 57).

MPH has been shown to have cognitive enhancing effects in healthy subjects (58, 59) and in patients with attention deficit hyperactivity disorder (ADHD), with a role in normalizing cerebral function in the latter (60–62). MPH improved inhibitory control, as measured by the Stop Signal task, in cocaine-dependent subjects in association with increased activation of the left DLPFC (63). Moreover, MPH has been shown to normalize cingulate activity in cocaine-dependent subjects during performance of a rewarded drug cue-reactivity task (64), as well as patients with ADHD during performance of cognitive control tasks (65, 66), including the color-word Stroop task (66). Although MPH modulated areas within the PFC associated with error-related processing in cocaine-dependent subjects performing the color-word Stroop task, it did not alter task performance (67).

MPH is an inhibitor of both the dopamine transporter and noradrenaline transporter (68–76). Inhibition of monoamine transporters by MPH leads to increased extracellular concentrations of dopamine in the PFC (77–80) and striatum (69, 81), and of noradrenaline in the PFC (77–80). The pharmacological effects of MPH are similar to those of cocaine and MA (82, 83). The overlap between the effects of MPH and those of MA suggests MPH may be an effective agonist replacement for MA dependence, and the use of MPH has been associated with positive early findings (51, 57). For example, MPH (54 mg/day) was superior to both aripiprazole (15 mg/day) and placebo for decreasing MA use in MA-dependent subjects (57). Although results from the randomized controlled trial that our sample of MA-dependent subjects were recruited from (the parent trial) showed no difference between MPH (Concerta^®^ ER; 54 mg/day) and placebo in the percentage of MA-positive urine samples, a higher retention rate was found in the MPH-treated MA-dependent group in comparison to that in the placebo-treated group (84).

The aim of this study was to investigate the behavioral and neural correlates of cognitive control using fMRI with the color-word Stroop task before and after an acute MPH (18 mg) challenge compared to placebo in active MA-dependent subjects and control subjects. To the best of our knowledge, this is the first fMRI study of cognitive control in active MA-dependent subjects, and the first to study MPH in MA dependence using fMRI. Consequently, the patterns of neural activations associated with the color-word Stroop task in active MA-dependent subjects, both at baseline and following MPH treatment, have not been previously reported and the effects of MPH treatment also remain unknown. Based on previous fMRI studies of cognitive control in abstinent MA-dependent subjects (3, 5), we hypothesized that our sample of active MA-dependent subjects would exhibit deficits in fMRI activation of frontal and cingulate brain regions during conditions of cognitive conflict with or without corresponding deficits in task performance. Since a previous study showed MPH (20 mg) administration did not cause behavioral changes in Stroop task performance in cocaine-dependent subjects (67), we did not expect the low dose of MPH (18 mg) used in the current study to change task performance during any of the Stroop conditions or to induce changes in neural activation during the congruent condition, since it does not require conflict resolution. However, we hypothesized that MPH would induce different neural activation patterns in active MA-dependent subjects relative to control subjects during the incongruent and Stroop effect conditions. Since MPH has been previously shown to modulate activity of areas within the cingulate cortex (64) and PFC (67), including the DLPFC (63) of cocaine-dependent subjects during tasks of cognitive control, we hypothesized that MPH administration would cause alterations in activation of the DLPFC and cingulate regions of MA-dependent subjects during Stroop conflict resolution. An exploratory whole-brain analysis method was employed to test these hypotheses, since there have been no prior fMRI studies of active MA-dependent subjects during tasks of cognitive control, nor have there been studies of the effects of MPH on the brains of MA-dependent subjects.

## Materials and Methods

### Subjects, drugs, and testing procedure

Fifteen adult subjects with a history of MA dependence and still actively using MA (four females; age 35.3 ± 7.0 years) were recruited from Community Alcohol and Drug Services in Point Chevalier, Auckland, New Zealand and 18 matched control subjects with no previous history of drug dependence (six females; age 31.1 ± 8.1 years) were recruited by word of mouth and advertisements (Table 1). MA-dependent subjects who were eligible for and interested in the current study were recruited from a randomized controlled trial of MPH compared to placebo in active MA-dependent subjects (the parent trial), which used percentage of MA-negative urine samples as its primary outcome measure (84). MA-dependent subjects were screened and diagnosed by a consultant psychiatrist using a structured clinical interview (SCID-I, Clinical Trials Version) (85). Data collected also included detailed questions regarding age at first use, route of administration, average amount of MA use per day, number of days used per week, and duration of regular use as well as self-rated level of use (light, regular, heavy). This was used to estimate the lifetime cumulative amount of MA used (Table 1). MA-dependent subjects also underwent physical examinations, and blood and urine testing to ensure health. They fulfilled the following inclusion criteria: (1) age between 18 and 46 years and of any ethnicity; (2) diagnosis of MA dependence according to DSM-IV criteria; current MA use was confirmed by qualitative urine drug tests (cut-off 300 μg/L); (3) urine toxicology screen testing for MA, cocaine, opiates, cannabis, and benzodiazepine compounds, which was negative for all except for MA and cannabis and no current or past history of other drug dependence, such as alcohol, cannabis, cocaine, opioids, or benzodiazepines; (4) taking no other prescribed medications, except for oral contraceptives and mild analgesics when required. Exclusion criteria were: (1) past or present Axis I psychiatric diagnosis (other than MA dependence, but including schizophrenia and major depression); (2) neurological, thyroid, renal, gastrointestinal, or cardiovascular disease; (3) clinically significant hepatic disease; (4) past or present illnesses known to affect cognition (e.g., stroke, traumatic brain injury, epilepsy, Parkinson’s disease, neurodegenerative disorders); (5) risk of suicide or violent behavior; (6) glaucoma; (7) Tourette’s disorder or tics; (8) in females, current pregnancy or lactation; (9) any contraindications to magnetic resonance imaging (MRI) (e.g., claustrophobia and implanted ferromagnetic objects).

**Table 1:** **Mean ± standard deviation (range) for demographic characteristics of subjects**.

	“Control MPH” subjects (*n* = 8)	“Control placebo” subjects (*n* = 10)	“MA MPH” subjects (*n* = 8)	“MA placebo” subjects (*n* = 7)
Age (years)	29.5 ± 7.5 (23–46)	32.3 ± 8.7 (18–44)	32.8 ± 7.5 (22–44)	38.3 ± 5.6 (28–46)
Gender (males/females)	5/3	7/3	4/4	7/0
Social drinking (*n*)	2	6	6	3
Regular nicotine use	0	0	7	6
Cannabis use	–	–	7	6
**MA USE VARIABLES**				
Route of administration (smoking/IV/both)	–	–	7/0/1	5/2/0
Age at first use (years)	–	–	23.3 ± 7.2 (12–34)	24.4 ± 7.2 (15–32)
Duration of use (years)	–	–	8.8 ± 2.7 (4–11)	13.0 ± 7.7 (2–25)
Amount of MA used per year (g)	–	–	138.7 ± 173.7 (23–520)	114.9 ± 107.2 (11–270)
Lifetime cumulative MA use (g)	–	–	1320.0 ± 1772.5 (98–5200)	1936.9 ± 2210.6 (23–5400)

*MA, methamphetamine; MPH, methylphenidate; IV, intravenous*.

Control subjects fulfilled the same inclusion/exclusion criteria as those with MA dependence except they were also excluded if they had a history of drug use.

Active MA-dependent subjects were randomly divided into two groups: eight subjects were assigned to the “MA MPH” group (four females; age 32.8 ± 7.5 years) and seven subjects were assigned to the “MA placebo” group (all males; age 38.3 ± 5.6 years) (Table 1). Control subjects were also randomly divided into two groups; 8 subjects were assigned to the “control MPH” group (three females; age 29.5 ± 7.5 years) and 10 subjects were assigned to the “control placebo” group (three females; age 32.3 ± 8.7 years) (Table 1).

Subjects underwent fMRI while performing the color-word Stroop task before (pre-drug scan) and approximately 1.5 h after (post-drug scan) receiving a single dose of their assigned medication. The capsules used for drug administration were identical in appearance; placebo capsules contained methylcellulose and MPH capsules contained one osmotic-controlled release oral delivery system (OROS) MPH (Concerta^®^ ER) tablet (18 mg). The 1.5 h delay between doses was chosen based on the OROS MPH producing a peak in plasma concentrations within the first 1–2 h following administration (86, 87). All procedures were approved by the Northern X Regional Ethics Committee of New Zealand (Ref: NTX/08/09/089) and subjects gave written informed consent prior to taking part in this study.

### The color-word stroop task

During the fMRI scan, the Stroop task stimuli were presented individually at the center of a screen located 3.5 m from the subject, and reflected into a mirror prism within the head coil. The stimuli were presented in bold Courier New style font, size 75 against a gray background, using E-Prime v2.0 (Psychological Software Tools, Pittsburgh, PA, USA). The task consisted of congruent, incongruent, neutral, and rest (fixation cross) blocks. The stimuli were four color-words (“RED,” “BLUE,” “GREEN,” and “YELLOW”) in matching font colors for the congruent condition (e.g., the word “
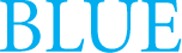
” displayed in blue font color) and non-matching font colors for the incongruent condition (e.g., the word “
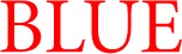
” displayed in red font color). Stimuli in the neutral condition were words matched with the color-words for length and frequency within the English language (“LOT,” “SHIP,” “KNIFE,” and “FLOWER”), and presented once in each of the four colors (e.g., the word “
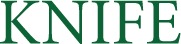
” displayed in green font color). During a single fMRI run, two congruent, two incongruent, and two neutral blocks were interspersed with seven rest blocks.

Subjects performed the task twice in the scanner, once before drug administration and once approximately 1.5 h after drug administration. The order was counterbalanced within and across subjects. Each task block lasted 40 s and consisted of 16 randomly selected stimuli, each with a duration of 2000 ms and a variable inter-stimulus interval of 400, 500, or 600 ms (average 500 ms). During task blocks, subjects were instructed to identify the font color of each word presented as quickly and accurately as possible, using two magnetic-compatible two-button response boxes held in each hand. For the red and blue target colors, subjects were to respond using their left middle and index fingers, respectively, and to respond to the green and yellow target colors, subjects were to press the right index and middle fingers, respectively. Accuracy and RT were recorded in E-Prime v2.0. During rest blocks, a black fixation cross was presented at the center of the screen for 16 s and subjects were instructed to take a break without closing their eyes. The task was approximately 6 min in duration. Subjects were trained outside the scanner on the correct finger positions for each target color and were required to achieve a minimum accuracy of 75% before proceeding to perform the task during fMRI.

In this paradigm, the Stroop effect was used as an index of response conflict in both task-related behavioral and functional brain activation analyses. The Stroop effect was computed as the difference in RT, accuracy, and fMRI brain activation between incongruent and congruent conditions.

### Magnetic resonance image acquisition/scanning parameters

Magnetic resonance imaging was conducted at the Centre for Advanced MRI, University of Auckland using a 1.5-T Siemens Magnetom Avanto scanner (Siemens Medical Solutions, Erlangen, Germany). Functional data were acquired along the AC–PC line with a ${\text{T}}_{2}^{*}$-weighted EPI sequence of 35 contiguous axial slices (TR = 3000 ms, TE = 50 ms, flip angle = 90°, FOV = 192 mm, matrix size = 64 × 64), yielding 3 mm^3^ isotropic voxels. The functional data were collected during a single run consisting of 118 volumes, after discarding the first three “dummy” volumes at the beginning of each session to allow for T_1_ signal equilibrium to be reached. Structural data were acquired with a high resolution T_1_-weighted anatomical magnetization-prepared rapid acquisition gradient-echo (MP-RAGE) sequence (144 slices 1.25 mm thick, TR = 2400 ms, TE = 3.61 ms, TI = 1000 ms, flip angle = 8°, FOV = 240 mm, matrix size = 144 × 192), yielding 1.25 mm^3^ isotropic voxel resolution.

### Data analyses

#### Behavioral data analysis

##### Pre-drug analysis: baseline group effects

Two-way repeated-measures analysis of variance (ANOVA) were conducted using STATA (v11.2), with condition (congruent and incongruent) and group (MA-dependent and control) as factors. Separate models were computed for RT and accuracy. Additionally, *post hoc* group difference contrasts were computed separately for each condition. For RT analyses, only trials for which correct responses were recorded during the scanning session were included.

##### Post-drug minus pre-drug analysis: group–drug effects

Two-way ANOVA analyses were conducted using STATA (v11.2) to investigate post-drug minus pre-drug differences with group (MA-dependent and control) and drug (MPH and placebo) as factors. Separate models were computed for accuracy and RT for each condition (congruent and incongruent) and for the Stroop effect. For RT analyses, only trials for which correct responses were recorded during the scanning session were included.

#### Functional magnetic resonance imaging data analysis

Statistical Parametric Mapping (SPM8) software (Wellcome Trust Centre for Neuroimaging, University College London, UK)^1^ was used for image processing and statistical whole-brain voxel-wise analyses.

Functional data were motion corrected, normalized to the Montreal Neurological Institute (MNI) space (88), resampled to 2 mm × 2 mm × 2 mm isotropic voxels and spatially smoothed using a Gaussian smoothing kernel with full-width at half-maximum of 8 mm × 8 mm × 8 mm.

Quality assurance was conducted by detecting artifactual volume outliers including those due to motion and de-weighting them using the Artifact Detection Tool (ART, Massachusetts Institute of Technology, Boston, MA, USA). For the analysis of each subject’s fMRI time-series, a first-level statistical model was constructed using a General Linear Model, including the three experimental conditions of interest: congruent, incongruent, and neutral. Each experimental block was convolved with the canonical hemodynamic response function. Rest blocks were not explicitly defined and were used as an implicit baseline. A high-pass filter cut-off of 128 s was used in an effort to remove slow signal drifts which were not task-related. Four contrasts were constructed for each subject; three *t*-contrasts testing the congruent, incongruent, and Stroop effect (incongruent–congruent) conditions, and the fourth contrast being an *F*-contrast across all conditions, used as a check of activity for quality assurance.

##### Pre-drug analysis: baseline group effects

The first analysis was conducted using the subject-level contrast images from the pre-drug scans to establish the neural correlates of the color-word Stroop task in active MA-dependent subjects in comparison to control subjects prior to drug treatment.

For within-group analyses, one-sample *t*-tests were conducted in both MA-dependent and control groups to reveal areas of activation across the three conditions of interest: congruent, incongruent, and Stroop effect. For between-group analyses, two-sample *t*-tests were conducted to reveal group differences in activation in the three conditions of interest. Significant clusters of fMRI activation were defined as clusters with at least 308 contiguous voxels attaining *p* < 0.005 based on analysis of functional neuroimages (AFNI)’s 3dClustSim program (89).

##### Post-drug minus pre-drug analysis: group–drug effects

In order to study the effects of MPH versus placebo in both groups, blood-oxygen-level-dependent (BOLD) activations from the pre-drug fMRI scans were subtracted from those in the post-drug fMRI scans. Specifically, pre-drug contrast images of interest (congruent, incongruent, and Stroop effect) were subtracted from their corresponding post-drug contrast images. This process resulted in one “post-drug minus pre-drug” contrast image per condition per subject. Group-level 2 × 2 (group × drug) between-subject ANOVAs were conducted using the subject-level “post-drug minus pre-drug” contrast images as inputs, to investigate group differences in fMRI activation across the three Stroop task conditions. *T*-contrasts were used to investigate group × drug interactions, within-group fMRI activation patterns and between-group differences in fMRI activation.

Significant clusters of fMRI activation were defined as clusters with at least 290 contiguous voxels attaining *p* < 0.005 based on AFNI’s 3dClustSim program (89).

Anatomical labeling was performed using a custom script within MatLab named peak_nii^2^ and the automated anatomical labeling (AAL) atlas (90).

## Results

### Demographics

There were no significant effects of group, drug, or group × drug interactions for age (Table 1). Chi-square tests were used to test differences in categorical variables; however, due to the small sample sizes, at least one cell had a count of 5 or less and the Fisher’s Exact Test (FET) statistic was reported. There were no group differences in gender (FET, *p* = 0.448) or alcohol use (FET, *p* = 0.084). However, there were significant group differences in tobacco smoking (FET, *p* < 0.0001) between active MA-dependent and control groups. The same is also true across the four sub-groups. Two-sample independent *t*-tests between the “MA MPH” and “MA placebo” groups revealed no significant group differences in cannabis use status (FET, *p* = 0.733) or MA use variables such as age at first use (*T*_13_ = −0.317, *p* = 0.756), duration of use (*T*_13_ = −1.478, *p* = 0.163), amount of MA used per year (*T*_13_ = 0.314, *p* = 0.759), and lifetime cumulative MA use (*T*_13_ = -0.600, *p* = 0.559) (Table 1).

### Behavioral results

#### Pre-drug analysis: baseline group effects

Accuracy and RT data by group and condition are presented in Table 2. Analysis of the mean percentage of trials that subjects performed correctly revealed a significant effect of condition (*F*_1,31_ = 5.493, *p* = 0.026 – congruent > incongruent), but no significant effect of group (*F*_1,31_ = 1.436, *p* = 0.240) or condition × group interaction (*F*_1,31_ = 1.652, *p* = 0.208).

**Table 2:** **Mean ± standard error for accuracy and response time during performance of the color-word Stroop task prior to drug administration (baseline group effects)**.

Measure	Controls	MA-dependent	*p*-Value
**ACCURACY (% CORRECT)**			
Congruent	96.6 ± 1.1	93.7 ± 1.2	0.0737
Incongruent	95.4 ± 1.1	89.7 ± 1.2	***0.0009***
Stroop effect – (congruent–incongruent)	1.2 ± 0.5	4.0 ± 2.3	0.2082
**REACTION TIME (MS)**			
Congruent	748.77 ± 18.28	883.50 ± 20.03	***<0.0001***
Incongruent	901.28 ± 18.28	989.27 ± 20.03	***0.0028***
Stroop effect – (incongruent–congruent)	152.50 ± 16.95	105.77 ± 36.84	0.2322

**p*-values are two-sided. Significant values are in bold italics*.

*MA, methamphetamine*.

For mean RTs, there were significant effects of condition (*F*_1,31_ = 45.363, *p* < 0.0001 – incongruent > congruent) and group (*F*_1,31_ = 4.916, *p* = 0.034 – MA-dependent > control), but no significant group × condition interaction (*F*_1,31_ = 1.485, *p* = 0.232).

*Post hoc* tests of each condition revealed that MA-dependent subjects were significantly slower than control subjects for both incongruent trials (989.27 ± 20.03 versus 901.28 ± 18.28 ms; mean ± standard error, *p* = 0.0028) and congruent trials (883.50 ± 20.03 versus 748.77 ± 18.28 ms; mean ± standard error, *p* < 0.0001) (Table 2). For the Stroop effect, MA-dependent subjects had lower RTs (105.77 ± 36.84 ms; mean ± standard error) than control subjects (152.50 ± 16.95 ms; mean ± standard error); however, this was not statistically significant (Table 2).

#### Post-drug minus pre-drug analysis: group–drug effects

There were no significant main effects or interactions of group or drug for any of the three conditions for accuracy (Table 3). Analysis of the mean RT revealed a significant main effect of group for the Stroop effect condition (*F*_1,29_ = 5.15, *p* = 0.031), but no significant effect of drug or group × drug interaction for RT during the Stroop effect condition and no significant main effects or interactions for RT during the congruent and incongruent conditions (Table 3). An increase in RT during the Stroop effect condition was observed in MA-dependent subjects post-drug compared to pre-drug administration (67.34 ± 26.10 ms; mean ± standard error) relative to controls (3.35 ± 19.24 ms; mean ± standard error). A trend was observed (*F*_1,29_ = 4.06, *p* = 0.053) for a decreased Stroop effect RT post-drug compared to pre-drug administration in all subjects who received MPH (3.10 ± 24.06 ms) relative to those who received placebo (60.05 ± 21.37 ms).

**Table 3:** **Mean ± standard error for post-drug minus pre-drug measures of accuracy and response time during performance of the color-word Stroop task (group–drug effects)**.

Measure	Controls		MA-dependent	
	MPH	Placebo	MPH	Placebo
**ACCURACY (% CORRECT)**				
Congruent	5.9 ± 3.5	0.3 ± 3.2	2.4 ± 3.5	2.6 ± 3.8
Incongruent	5.5 ± 4.5	−7.8 ± 4.0	1.0 ± 4.5	−1.9 ± 4.8
Stroop effect – (congruent–incongruent)	0.4 ± 3.6	8.1 ± 3.2	1.4 ± 3.6	4.4 ± 3.9
**RESPONSE TIME (ms)**				
Congruent	−62.51 ± 28.32	−56.60 ± 25.33	−88.57 ± 28.32	−110.75 ± 30.28
Incongruent	−100.71 ± 41.66	−20.00 ± 37.26	−44.16 ± 41.66	−17.19 ± 44.54
Stroop effect – (incongruent–congruent)	−38.21 ± 30.97	36.60 ± 27.70	44.41 ± 30.97	93.55 ± 33.11

*Values represent post-drug minus pre-drug measures*.

*MA, methamphetamine; MPH, methylphenidate*.

### Functional magnetic resonance imaging results

#### Pre-drug analysis: baseline group effects

##### Within-group results

For the congruent and incongruent conditions, robust activations were observed in the calcarine fissure, inferior occipital gyrus, middle occipital gyrus (MOG), fusiform gyrus, inferior parietal lobule (IPL), superior parietal gyrus (SPG), precentral gyrus, post central gyrus, and supplementary motor area, of both MA-dependent and control groups (*p* < 0.05 cluster-corrected). Additionally, activations in the control group were observed in the supramarginal gyrus, inferior temporal gyrus (ITG), superior temporal gyrus (STG), inferior frontal gyrus (IFG), and superior occipital gyrus (SOG) in response to the congruent condition only, and in the superior frontal gyrus (SFG) during the incongruent condition only (*p* < 0.05 cluster-corrected). Control subjects showed activation in the middle frontal gyrus (MFG) during both the congruent and incongruent conditions (*p* < 0.05 cluster-corrected). In contrast, activations in the MA-dependent group were observed in the anterior cingulate gyrus (ACG), and middle cingulate gyrus (MCG) during the congruent condition only, the SOG, precuneus, and ITG during the incongruent condition only and the supramarginal gyrus, IFG, MFG, SFG, and middle temporal gyrus (MTG) in response to both congruent and incongruent conditions (*p* < 0.05 cluster-corrected).

Control subjects exhibited no significant activations corresponding to the Stroop effect. However, increased activation of the IPL was observed in the MA-dependent group (*p* < 0.05 cluster-corrected).

##### Between-group results

There were no differences in fMRI activation between MA-dependent and control groups during the congruent condition (Table 4). However, the two groups significantly differed during the incongruent and Stroop effect conditions, with the MA-dependent subjects exhibiting greater fMRI activation than control subjects (Table 4).

**Table 4:** **Whole-brain two-sample *t*-test analyses of group differences in blood-oxygen-level-dependent activation during the three Stroop conditions**.

Condition (*t*-contrast)	Cluster size (voxels)	Regions of peak voxels	HS	MNI co-ordinates (mm)			Peak *t*-statistic
				*x*	*y*	*z*	
Congruent	Control > MA, *p* < 0.05 cluster-corrected					
	Nil	Nil	Nil	Nil	Nil	Nil	Nil
	MA > control, *p* < 0.05 cluster-corrected					
	Nil	Nil	Nil	Nil	Nil	Nil	Nil
Incongruent	Control > MA, *p* < 0.05 cluster-corrected					
	Nil	Nil	Nil	Nil	Nil	Nil	Nil
	MA > control, *p* < 0.05 cluster-corrected					
	1743	Superior frontal gyrus	R	28	4	60	5.73
				21	14	58	5.69
				20	6	50	5.13
		Middle frontal gyrus	R	30	10	46	3.84
Stroop effect	Control > MA, *p* < 0.05 cluster-corrected					
	Nil	Nil	Nil	Nil	Nil	Nil	Nil
	MA > control, *p* < 0.05 cluster-corrected					
	1680	Inferior parietal lobule	R	48	−48	52	4.62
				54	−40	56	4.27
	319	Inferior parietal lobule	L	−46	−54	58	3.81
				−54	−42	48	3.31

*Significant clusters of activation were defined as clusters with at least 308 contiguous voxels attaining *p* < 0.005. Co-ordinates represented are in MNI space. HS, hemisphere; L, left; R, right*.

**Figure 1:**
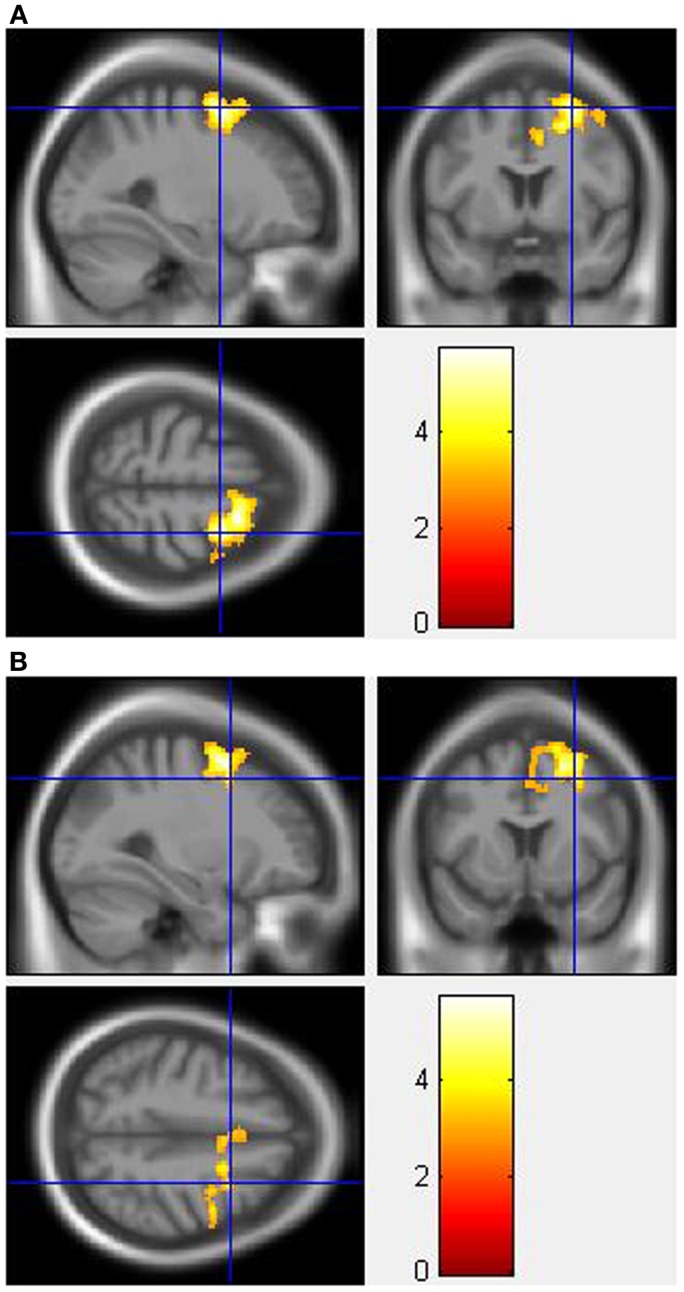
**Whole-brain two-sample *t*-test results for the incongruent condition showing pre-drug MA-dependent > control group differences in activation of: (A) the right superior frontal gyrus [peak voxel MNI co-ordinates (mm): (28, 4, 60); *T*_31_ = 5.73, *p* < 0.05 cluster-corrected], and (B) the right middle frontal gyrus [peak voxel MNI co-ordinates (mm): (30, 10, 46); *T*_31_ = 3.84, *p* < 0.05 cluster-corrected]**. The scale represents the color (from red to yellow) of the cluster corresponding to the increasing *t*-statistic. The structural image represents the IXI550 average normal brain which has been registered to the MNI152 space with corresponding inferior–superior co-ordinates.

During the incongruent condition, MA-dependent subjects showed greater activation than control subjects in the right SFG (Figure 1A) and right MFG (Figure 1B) (*p* < 0.05 cluster-corrected).

**Figure 2:**
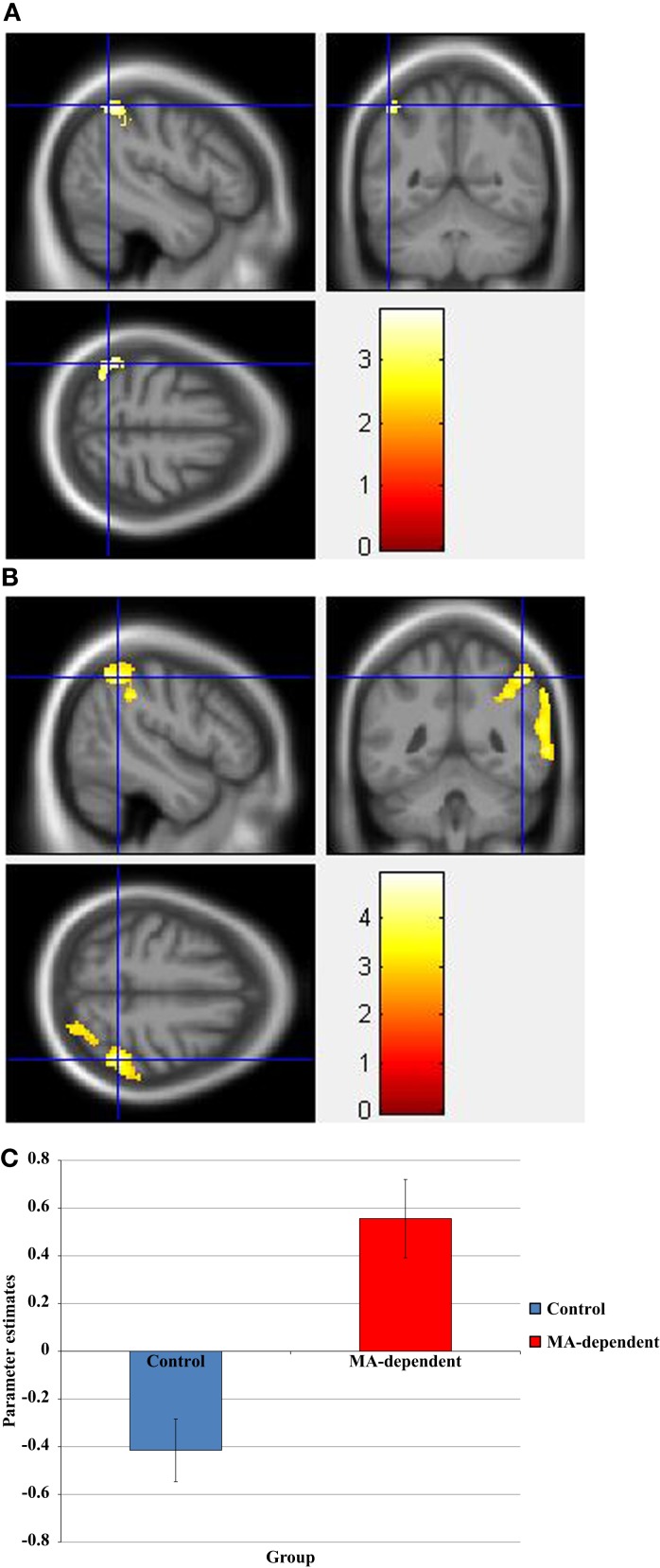
**Whole-brain two-sample *t*-test results for the Stroop effect condition showing pre-drug MA-dependent > control group differences in activation of: (A) the left inferior parietal lobule [peak voxel MNI co-ordinates (mm): (−46, −54, 58); *T*_31_ = 3.81, *p* < 0.05 cluster-corrected], (B) the right inferior parietal lobule [peak voxel MNI co-ordinates (mm): (48, −48, 52); *T*_31_ = 4.62, *p* < 0.05 cluster-corrected], and (C) Plot of the mean ± standard error parameter estimates representing the percentage blood-oxygen-level-dependent signal change within the right inferior parietal lobule**. The scale represents the color (from red to yellow) of the cluster corresponding to the increasing *t*-statistic. The structural image represents the IXI550 average normal brain which has been registered to the MNI152 space with corresponding inferior–superior co-ordinates.

For the Stroop effect, MA-dependent subjects exhibited greater activation than control subjects of the left (Figure 2A) and right IPL (Figures 2B,C) (*p* < 0.05 cluster-corrected).

#### Post-drug minus pre-drug analysis: group–drug effects

##### Within-group results

During the congruent condition, there were no significant changes in fMRI activation post- compared to pre-drug administration in any of the four groups.

For the incongruent condition, the “control MPH” group showed increased activation post- compared to pre-MPH administration of the left and right IPL, left SPG, left IFG, left MFG, right STG, right MTG, right SOG, left and right MOG, and left and right MCG (*p* < 0.05 cluster-corrected). The “control placebo” group exhibited increased activation post- compared to pre-placebo administration of the right MTG, right STG, and right MOG (*p* < 0.05 cluster-corrected). Whereas the “MA MPH” group showed no significant change in activation post- compared to pre-MPH administration during the incongruent condition, the “MA placebo” group exhibited increased activation of the left and right MOG, left and right SOG, left and right cuneus, left SPG, and right precuneus (*p* < 0.05 cluster-corrected).

During the Stroop effect condition, control and MA-dependent subjects showed no significant change in fMRI activation following placebo administration. However, following MPH administration, the “control MPH” group showed increased activation of the left MOG, left IPL, left SPG, right MCG, left ACG, left SFG, left IFG, and right supramarginal gyrus, and the “MA MPH” group exhibited increased activation of the right IFG and right MFG (*p* < 0.05 cluster-corrected).

##### Between-group results

For the congruent condition, there were no significant group differences in fMRI activation post- compared to pre-drug administration (Table 5).

**Table 5:** **Whole-brain 2 × 2 between-subject ANOVA analyses of group differences in blood-oxygen-level-dependent activation during the three Stroop conditions**.

Condition	Cluster size (voxels)	Regions of peak voxels	HS	MNI co-ordinates (mm)			Peak *t*-statistic
				*x*	*y*	*z*	
Congruent	Nil	Nil	Nil	Nil	Nil	Nil	Nil
Incongruent	“MA placebo” > “MA MPH,” *p* < 0.05 cluster-corrected						
	936	Superior occipital gyrus	L	−20	−88	44	4.54
			R	17	−92	34	3.53
		Superior parietal gyrus	L	−8	−82	52	4.49
		Middle occipital gyrus	R	40	−81	27	3.65
	“Control MPH” > “MA MPH,” *p* < 0.05 cluster-corrected						
	293	Inferior parietal lobule	R	56	−36	54	3.34
Stroop effect	“Control MPH” > “MA MPH,” *p* < 0.05 cluster-corrected						
	332	Middle occipital gyrus	L	−32	−82	30	4.18
	360	Middle occipital gyrus	R	45	−80	24	3.79

*Significant clusters of activation were defined as clusters with at least 290 contiguous voxels attaining *p* < 0.005. Co-ordinates represented are in MNI space. HS, hemisphere; L, left; R, right*.

**Figure 3:**
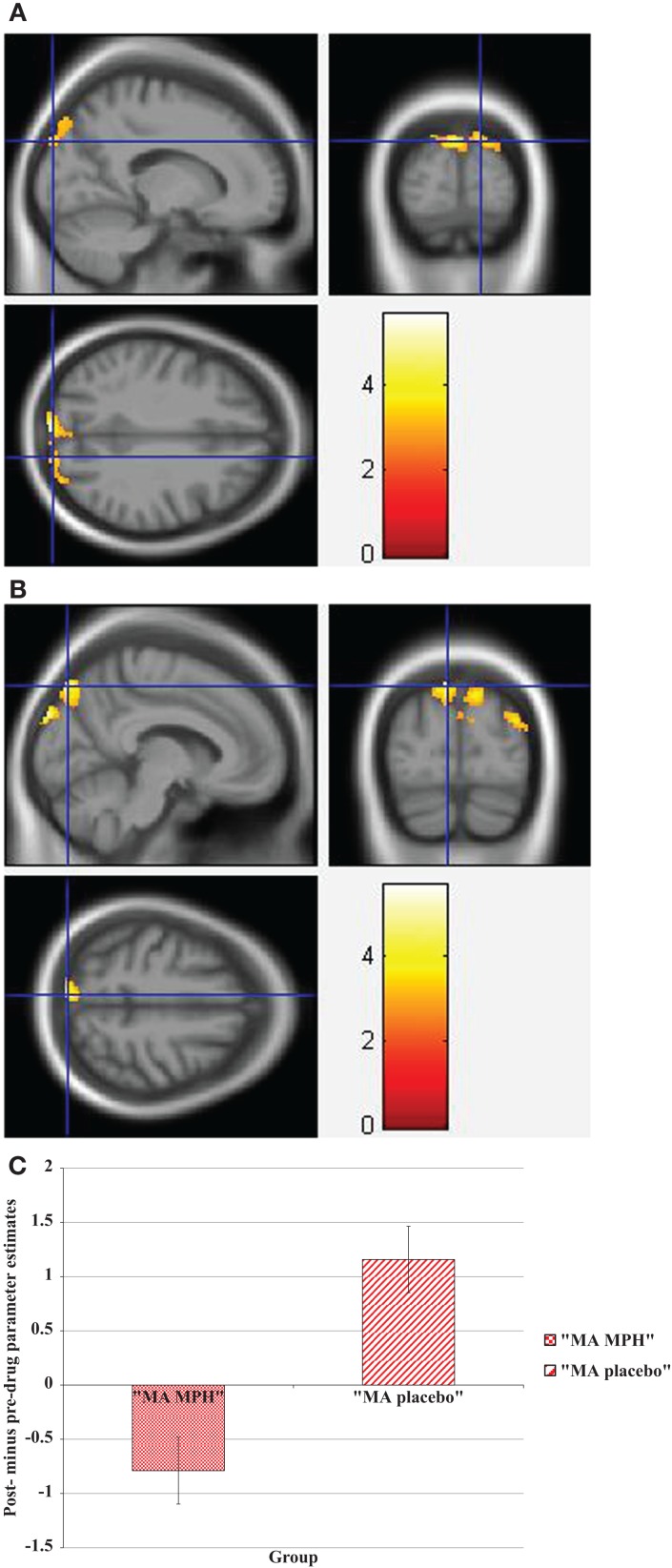
**Whole-brain 2 × 2 between-subject ANOVA results for the incongruent condition showing post- minus pre-drug “MA placebo” > “MA MPH” group differences in activation of: (A) the right superior occipital gyrus [peak voxel MNI co-ordinates (mm): (17, −92, 34); *T*_29_ = 3.53, *p* < 0.05 cluster-corrected], (B) the left superior parietal gyrus [peak voxel MNI co-ordinates (mm): (−8, −82, 52); *T*_29_ = 4.49, *p* < 0.05 cluster-corrected], and (C) Plot of the mean ± standard error parameter estimates representing the percentage blood-oxygen- level-dependent signal change post- compared to pre-drug administration within the left superior parietal gyrus**. The scale represents the color (from red to yellow) of the cluster corresponding to the increasing *t*-statistic. The structural image represents the IXI550 average normal brain which has been registered to the MNI152 space with corresponding inferior–superior co-ordinates.

**Figure 4:**
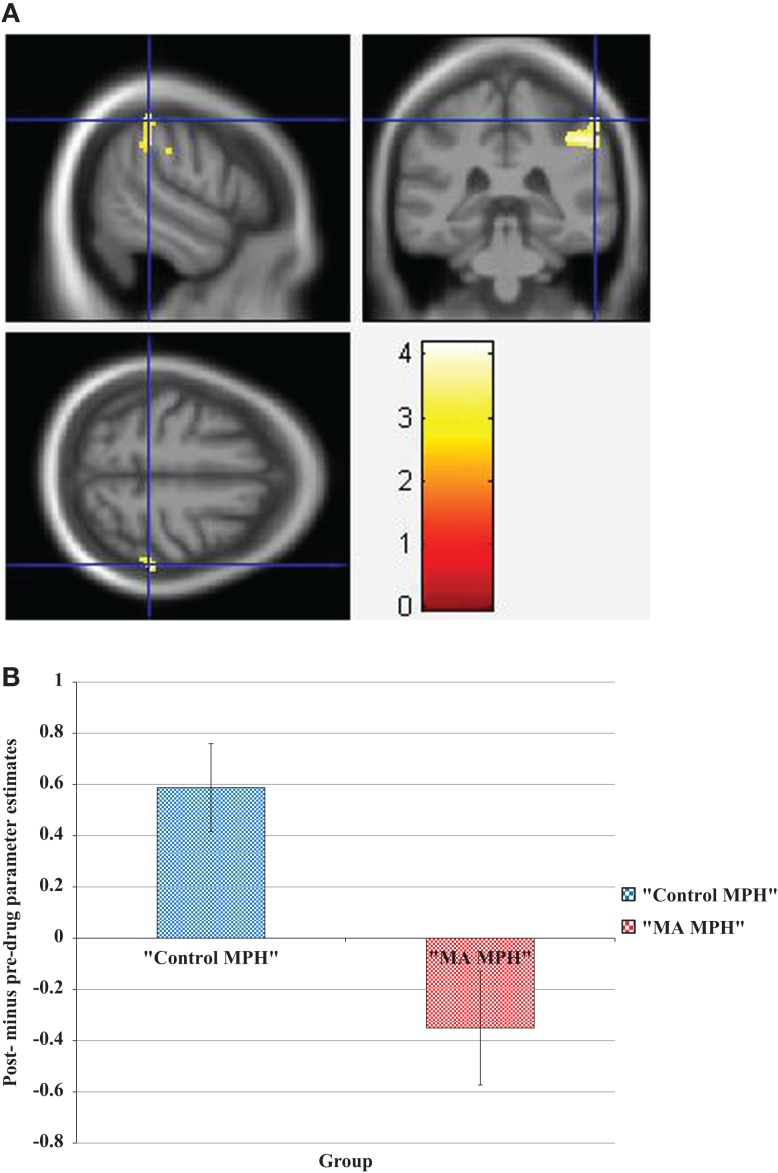
**(A) Whole-brain 2 × 2 between-subject ANOVA results for the incongruent condition showing post- minus pre-drug “Control MPH” > “MA MPH” group differences in activation of the right inferior parietal lobule [peak voxel MNI co-ordinates (mm): (56, −36, 54); *T*_29_ = 3.34, *p* < 0.05 cluster-corrected], and (B) Plot of the mean ± standard error parameter estimates representing the percentage blood-oxygen-level-dependent signal change post- compared to pre-drug administration within the right inferior parietal lobule**. The scale represents the color (from red to yellow) of the cluster corresponding to the increasing *t*-statistic. The structural image represents the IXI550 average normal brain which has been registered to the MNI152 space with corresponding inferior–superior co-ordinates.

During the incongruent condition, there was a significant group × drug interaction, whereby the “MA placebo” group showed increased activation post- compared to pre-drug administration relative to the “MA MPH” group of the left and right SOG (Figure 3A), right MOG, and left SPG (Figures 3B,C) (*p* < 0.05 cluster-corrected) (Table 5). Following MPH administration, the “control MPH” group showed increased activation compared to the “MA MPH” group of the right IPL (Figures 4A,B) (*p* < 0.05 cluster-corrected) (Table 5).

**Figure 5:**
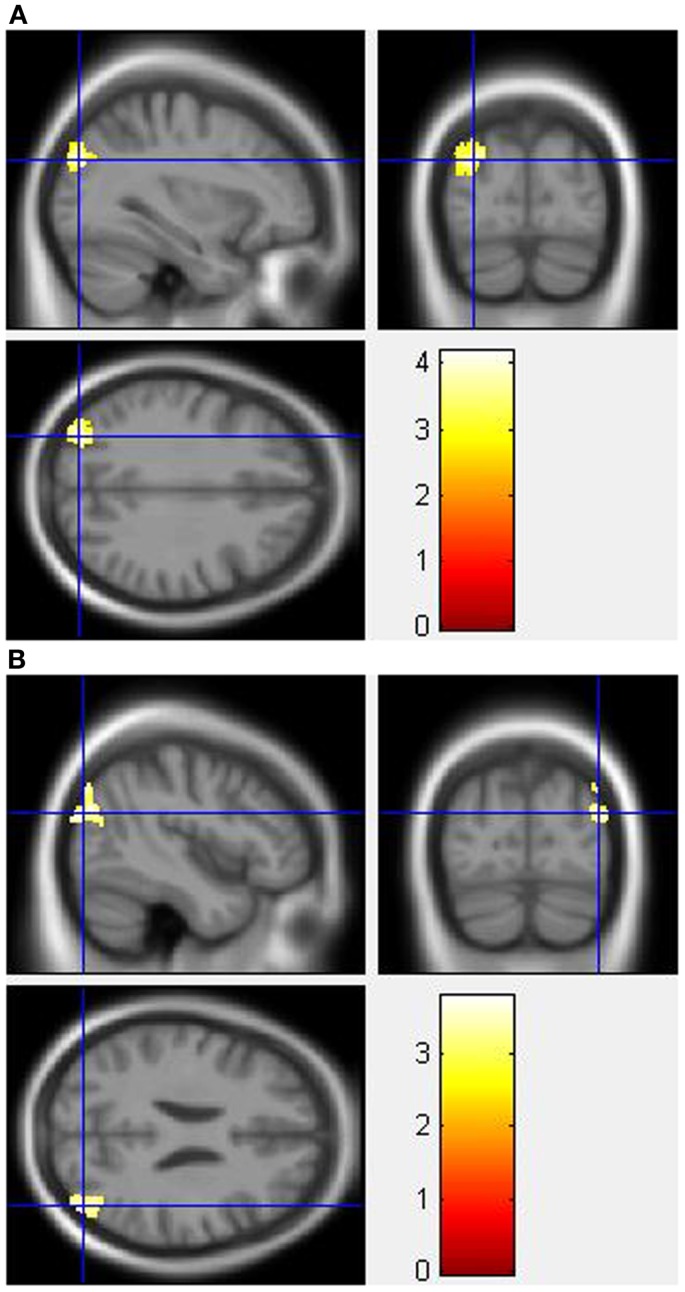
**Whole-brain 2 × 2 between-subject ANOVA results for the Stroop effect condition showing post- minus pre-drug “control MPH” > “MA MPH” group differences in activation of: (A) the left middle occipital gyrus [peak voxel MNI co-ordinates (mm): (−32, −82, 30); *T*_29_ = 4.18, *p* < 0.05 cluster-corrected], and (B) the right middle occipital gyrus [peak voxel MNI co-ordinates (mm): (45, −80, 24); *T*_29_ = 3.79, *p* < 0.05 cluster-corrected]**. The scale represents the color (from red to yellow) of the cluster corresponding to the increasing *t*-statistic. The structural image represents the IXI550 average normal brain which has been registered to the MNI152 space with corresponding inferior–superior co-ordinates.

During the Stroop effect condition, the “control MPH” group showed increased activation post- compared to pre-drug administration relative to the “MA MPH” group of the left (Figure 5A) and right MOG (Figure 5B) (*p* < 0.05 cluster-corrected) (Table 5). No other group comparisons exhibited significant differences in fMRI activation during the Stroop effect condition.

## Discussion

To the best of our knowledge, this is the first fMRI study to investigate the effects of MPH on cognitive control in active MA-dependent subjects in comparison to control subjects. Due to the small sample sizes used, the results of this study are preliminary and future studies with larger sample sizes are warranted to validate these results.

### Pre-drug analysis: Baseline group effects

#### Behavioral results

Consistent with previous literature, there were differences in RT but not accuracy during performance of the Stroop task between MA-dependent and control subjects prior to drug administration (5, 8, 31). Analyses of both accuracy and RT showed a significant main effect of condition, whereby both groups showed the typical Stroop interference effect with lower accuracy and longer RTs during the incongruent condition relative to the congruent condition. With respect to RT, there was also a significant main effect of group, whereby MA-dependent subjects exhibited slower responses than control subjects across both congruent and incongruent conditions. Longer RTs may reflect slower motor responses in MA-dependent subjects in comparison to healthy control subjects, as has been previously reported in substance-use disorders (91), and more specifically in stimulant dependence (92).

There were no statistically significant group differences in RT corresponding to the Stroop effect (incongruent RT minus congruent RT). However, the MA-dependent group displayed a lower mean RT corresponding to the Stroop effect in comparison to control subjects. This non-significant effect may have resulted from a smaller difference amongst MA-dependent subjects between congruent and incongruent RTs.

Although there have been studies which reported no differences in Stroop performance between abstinent MA-dependent individuals and control subjects (8, 93), our RT results do not replicate findings from several other studies that reported behavioral differences during the Stroop task in abstinent (5, 31) and active MA-dependent individuals (28, 29). For example, in cognitive studies of active MA-dependent individuals using neuropsychological tests, Simon and colleagues observed no group differences in performance during Stroop words, Stroop colors, or Stroop interference scores (28, 29). However, they showed worse performance on the Stroop color-words (equivalent to the incongruent condition of our version of the Stroop task) amongst active MA-dependent users (28, 29). The combination of normal performance on Stroop words and colors and worse performance on the Stroop color-words has been previously thought to be indicative of generalized brain atrophy (29, 94). The discrepancy in our findings from those of Simon and colleagues’ (28, 29) may be attributed to the different versions of the Stroop task used, which may not be directly comparable. The lack of significant difference in mean RT for the Stroop effect, combined with the absence of a condition × group interaction in RT or accuracy in the current study, suggest that MA-dependent subjects were no worse than control subjects in their behavioral Stroop interference scores. Hence, the behavioral results from the current study cannot be used to predict generalized brain atrophy. This is in accordance with the results of our voxel-based morphometry analysis of the same subject samples, which showed no differences in cortical GM volumes between active MA-dependent and control subjects (95).

#### fMRI results

In response to the Stroop effect condition, MA-dependent subjects showed significant BOLD activations within the inferior parietal cortex, while control subjects did not exhibit any change in fMRI activation. Although control subjects appeared to have a larger behavioral Stroop effect than MA-dependent subjects, this was not statistically significant. Therefore, the absence of change in neural activation in response to the Stroop effect for control subjects may suggest the task was not difficult enough for this group of subjects. For example, the task may require more blocks to elicit a brain response to the Stroop effect condition in healthy control subjects. Nonetheless, behavioral and fMRI activation changes observed in the MA-dependent group during both the incongruent and Stroop effect conditions suggest the task was of sufficient difficulty to elicit a response in MA-dependent subjects.

Increased BOLD activation of the PFC and parietal cortex was observed in both control and MA-dependent groups during the congruent and incongruent conditions of the color-word Stroop task. This finding of increased activation of prefrontal and parietal regions is opposite to our hypothesis that MA-dependent subjects would exhibit deficits in fMRI activation of frontal regions. Since this hypothesis was based on previous studies of MA-dependent subjects who were abstinent, it is possible that the discrepancy in findings may be attributed to the active user status of our sample of MA-dependent subjects. Successful conflict resolution during the Stroop task is expected to involve activity of frontal and superior parietal cortices, which are implicated in top-down stimulus biasing (46). The role of prefrontal and parietal regions in tasks involving cognitive control, particularly response inhibition, is well-established in healthy individuals (32, 33, 37–39, 42, 45, 46, 96, 97) and those with drug dependence (5, 8). In chronic MA-dependent subjects, abnormalities in regional cerebral glucose metabolism have been observed in the PFC, ACC, and parietal cortex (98–100).

Previous studies of cognitive control have documented the ACC (3, 5, 8, 34, 36, 37, 40–43) and DLPFC (5, 8, 34–39), which anatomically spans the SFG, IFG, and MFG (22, 101) as the primary neural substrates of conflict monitoring and cognitive control, respectively. The cingulate cortex was activated in MA-dependent subjects only when performing the congruent condition, during which they showed increased activation of the MCG. Activation of the cingulate cortex during the congruent and not the incongruent condition is somewhat unusual; as the cingulate cortex, specifically the ACC, has been generally implicated during the performance of incongruent conditions (8, 37) or in response to the Stroop effect (5), suggesting a role in performance monitoring (37). Although previous fMRI studies using the Stroop task in MA-dependent subjects found significant group differences in activation of the ACC during the incongruent condition, they were conducted in abstinent subjects (5, 8). Moreover, one of these studies did not investigate group differences in whole-brain activation and used regions of interest with specific hypotheses surrounding the ACC and PFC (8). Therefore, previous findings from fMRI studies using the Stroop task in abstinent subjects may not be directly comparable with our results. It is possible that active MA-dependent subjects do not need to activate the ACC during the Stroop conflict conditions.

The DLPFC has been shown to be active during color-naming conditions, confirming a role in the implementation of control (37). Using fMRI, the DLPFC has also been shown to be active in response to successful inhibition of unpredictable No-go events during a “sustained attention to response” task (102) and during the Stroop task (33). Activation of the DLPFC was observed in the current study, specifically in the SFG and MFG of MA-dependent subjects and the MFG of control subjects in response to both congruent and incongruent conditions. A significant between-group difference was found whereby MA-dependent subjects showed increased activation of the right SFG and MFG in comparison to control subjects during the incongruent condition. Increased SFG activation has been previously reported following successful conflict adaptation, corresponding with increased cognitive control in healthy individuals (35). The MFG is an important part of the cerebral circuit that underlies top-down control processes such as cognitive control, and has been specifically implicated in cognitive control during conflict adaptation (35, 103). In a previous study, abstinent cocaine-dependent subjects exhibited increased activation within the DLPFC, specifically the MFG, during conflict conditions of the color-word Stroop task in comparison to healthy control subjects (67). It is possible that MA-dependent subjects needed to recruit additional neural resources within the DLPFC in order to perform the task correctly and to the same level of efficiency as control subjects.

Increased activation of the IFG was also observed in the MA-dependent group during both congruent and incongruent conditions and in the control group during the congruent condition only. The IFG has been implicated in inhibitory control (104) and in the exertion of self-control through modulation by the DLPFC (105). Patients with lesions in the right IFG have impaired inhibitory control (106), and neuroimaging studies have shown that the IFG is involved in tasks requiring response inhibition such as the Go/No-go task (107, 108).

Both MA-dependent and control subjects activated the IPL and SPG within the parietal cortex during the incongruent condition. However, only MA-dependent subjects activated the IPL during the Stroop effect condition, resulting in a significant between-group difference in fMRI activation in this region. Both the incongruent and Stroop effect conditions present subjects with a stimulus-based conflict, requiring the subject to respond to word color, an unusual task-relevant stimulus feature and inhibit the word reading response, a prepotent response (26, 27). Stroop conflict resolution is thought to occur via a stimulus-biasing strategy (103, 109, 110), which involves modulation of activity in the parietal cortex (46). The parietal cortex is thought to be the origin of top-down signals (received from the PFC) that initiate selective attentional bias in favor of the task-relevant feature by amplifying visual processing toward it (111–114). The superior parietal cortex is involved in Stroop conflict resolution in healthy humans (46), which corresponds well with both control and MA-dependent subjects activating the SPG during the incongruent condition. In healthy subjects, response inhibition during Stroop conflict conditions has also been associated with fMRI activity in the inferior parietal cortex, which encompasses the IPL (32, 33). This process is also thought to result from biasing toward processing of the task-relevant stimulus feature involving similar top-down modulation from the PFC to the inferior parietal cortex (32, 115). The IPL is known to be involved in other tasks requiring response inhibition including the Simon task (33), Go/No-go task (97, 116, 117), and the Stop paradigm task (117). Although parietal regions have not been previously implicated in fMRI studies of MA-dependent subjects during cognitive control, increased IPL activation was reported in recently abstinent MA-dependent subjects with lower error rates, and during the most unpredictable condition while performing the two-choice prediction task (6). Our results suggest that MA-dependent subjects who are active users require recruitment of additional neural resources in the inferior parietal cortex for Stroop conflict resolution with similar behavioral efficiency to control subjects.

During the congruent and incongruent conditions of the Stroop task, both groups demonstrated robust activations of vision-related regions including the calcarine fissure and occipital cortex. The visual pathways including the occipital cortex are not typically associated with the cognitive control aspect of the Stroop task. However, during the Stroop task, subjects are instructed to respond to the ink color of a presented word (task-relevant stimulus) and inhibit their prepotent response to read the word (task-irrelevant stimulus) (26, 27). In order to achieve cognitive control, visual pathways may be activated in an effort to bias selective attention toward task-relevant stimulus features and distinguish them from task-irrelevant distracter features of the stimulus, leading to amplified neural processing of target (task-relevant) stimulus features (103).

### Post-drug minus pre-drug analysis: Group–drug effects

#### Behavioral results

Behaviorally, subjects did not differ in task accuracy post-drug compared to pre-drug administration. For the Stroop effect condition, MA-dependent subjects exhibited significantly longer RTs (higher interference scores) post-drug compared to pre-drug administration relative to control subjects, irrespective of drug treatment. However, this effect was more pronounced in placebo-treated MA-dependent subjects than those treated with MPH. Higher interference scores post- compared to pre-drug administration in MA-dependent subjects may be due to increased distractibility toward the end of the study session in comparison to the start of the session. Although acutely, MA improves attention and concentration (118), it has been clinically observed to cause increased distractibility (31). Furthermore, most of our MA-dependent subjects appeared distractible, despite scoring over 75% on the practice version of the Stroop task outside the scanner. For example, some subjects showed difficulty following conversation, while others found it challenging to follow instructions. This observation was thought to be worse during the second half of the study session than the first half. However, the increase in distractibility may have been tempered in the “MA MPH” group as we observed smaller, but not significantly different increases in Stroop effect RT post- compared to pre-drug administration relative to the “MA placebo” group.

Although there were no significant drug effects for mean RT, a trend was observed for a decreased Stroop effect RT in all subjects who received MPH compared to those who received placebo. This trend suggests that MPH administration caused improvement in task performance, manifested by a decreased interference score (Stroop effect RT) in all subjects, irrespective of group membership.

Several studies in children with ADHD showed improved performance following MPH administration during the color-word Stroop task (119) and the Go/No-go task (inhibitory control) (120) in both healthy children and those with ADHD, and during a rewarded continuous performance task (attention but not impulsiveness errors) in children with ADHD (121). MPH has also been shown to improve response inhibition in healthy adult subjects, with specific improvements in stop signal RT and RT variability during performance of the stop signal inhibition task, without changing overall speed (122). However, there have been studies that reported no change in Stroop task performance following MPH administration in patients with ADHD (123) or those with cocaine-dependence and their comparison subjects (67). Nonetheless, our study showed a trend for improved task performance in active MA-dependent subjects and their comparison subjects following MPH administration relative to placebo. Based on this trend, we cannot sustain our hypothesis that acute low-dose MPH (18 mg) administration would not change Stroop task performance efficiency, and studies using higher doses of MPH may be needed to come to any definitive conclusion.

#### fMRI results

In accordance with our hypothesis, there were no within- or between-group differences in fMRI activation post- compared to pre-drug administration during the congruent condition. Since the congruent condition does not present cognitive conflict, response inhibition is not required and therefore an acute dose of MPH would not be expected to induce a difference in task performance or neural activation.

There is an established role for the DLPFC (37), including the IFG (104, 106), MFG (8, 35, 103), and SFG (35) in conflict resolution and cognitive control. MPH administration resulted in increased activation of the left IFG and MFG during the incongruent condition and the left IFG and SFG during the Stroop effect condition in “control MPH” subjects; an effect that was not observed in the other three groups. The effects of MPH on the DLPFC are well-known; it has been shown to cause increased regional cerebral blood flow in the bilateral DLPFC in children with ADHD who were previously treatment-naïve (124). The IFG and MFG are known to be activated in response to the incongruent condition of the color-word Stroop task in healthy subjects and those with cocaine-dependence treated with MPH and placebo (67). MPH is also known to increase prefrontal activation equally in children with ADHD and healthy matched controls during the Go/No-go task (120). Following an acute dose of MPH, healthy control children showed increased activation of the right IFG in comparison to children with ADHD during a task of vigilant attention (121). However, this may not have been specific to MPH, as control children also exhibited increased activation of right IFG relative to those with ADHD following placebo (121). MPH has also been shown to cause an increase in activation of the MFG in healthy subjects performing tasks of visual attention and working memory, which was positively correlated with increasing cognitive load of the working memory task (125).

Although “MA MPH” subjects did not exhibit differences in BOLD activation within the DLPFC post- compared to pre-MPH administration during the congruent and incongruent conditions, there was a larger difference in activation of the IFG and MFG within the right DLPFC of these subjects between the incongruent and congruent conditions. This difference in activation post- compared to pre-MPH administration resulted in an increase in right DLPFC activation corresponding with the Stroop effect, which was accompanied by an increased Stroop effect RT (interference score). However, it is worth noting that the increase in Stroop effect RT (incongruent RT–congruent RT) resulted from a smaller improvement/reduction in RT for the incongruent condition compared to the congruent condition post- relative to pre-MPH administration (Table 3). MPH administration has been shown to modulate neural activation within the DLPFC of cocaine-dependent subjects during tasks of cognitive control (63). Therefore, taken together, increased RT and right DLPFC activation post- compared to pre-MPH administration during the Stroop effect condition, suggest an MPH-facilitated recruitment of additional neural resources within the DLPFC of MA-dependent subjects for Stroop conflict resolution. This finding supports our hypothesis that MPH administration would cause alterations in activation of the DLPFC of MA-dependent subjects during Stroop conflict resolution.

Between-group differences in fMRI activation were reported mainly within the parietal–occipital cortices, with “MA MPH” subjects exhibiting a greater decrease in activity within these regions during the incongruent condition compared to “MA placebo” and “control MPH” groups, and within the occipital cortex during the Stroop effect condition compared to the “control MPH” group.

Results from the pre-drug analysis of baseline group effects showed increased activation of parietal regions of MA-dependent subjects compared to control subjects corresponding with the Stroop effect condition. The parietal cortex plays an important role in cognitive control, particularly response inhibition in healthy subjects (32, 33, 39, 42, 45, 46, 96, 97). As mentioned earlier, Stroop conflict is thought to be resolved by a stimulus-biasing strategy (103, 109, 110) modulated by activity in the parietal cortex (46). Hence, it was suggested that active MA-dependent subjects require recruitment of additional neural resources within the parietal cortex for successful conflict resolution during conflict conditions. Results from the post-drug minus pre-drug analysis of group–drug effects showed increased activation within the parietal cortex of “control MPH” during the incongruent and Stroop effect conditions, an effect which has been previously reported in healthy subjects (46). Similarly to our baseline group results, increased activation within the parietal cortex of MA-dependent subjects post- compared to pre-placebo administration was also observed during the incongruent condition; however, the opposite effect was observed in MA-dependent subjects who were treated with MPH. In comparison to “MA placebo” subjects, “MA MPH” subjects showed decreased activation of the left SPG during the incongruent condition post-compared to pre-drug administration. Moreover, in comparison to “control MPH” subjects, ”MA MPH” subjects exhibited decreased activation of the right IPL during the incongruent condition post- compared to pre-drug administration. These findings suggest that MPH administration caused decreases in activation within the left superior and right inferior parietal cortex of MA-dependent subjects during the incongruent condition. The incongruent condition presents the subject with a stimulus-based conflict, whereby competition exists between two information sources: the task-relevant stimulus feature (the word color) and the task-irrelevant, but more automatic stimulus feature (word reading) (26, 27). It is possible that without treatment, active MA-dependent subjects exhibit a lowered threshold at which incongruent stimuli present sufficient conflict to activate the parietal cortex. In patients with ADHD, MPH treatment was shown to normalize an otherwise raised threshold at which task-related default mode network deactivation occurred, to achieve a pattern similar to that of healthy controls (126). Similarly, acute MPH treatment in active MA-dependent subjects may normalize the threshold at which incongruent stimuli present sufficient conflict to activate the parietal cortex, thereby decreasing the activity of this region (Figure 4B).

All subjects showed increased activation within the occipital cortex post- compared to pre-drug administration during the incongruent condition, with the exception of “MA MPH” subjects. However, only “control MPH” subjects showed an increase in occipital activation during the Stroop effect condition following MPH administration. These changes in activation resulted in increased activation of the occipital regions of “MA placebo” subjects compared to “MA MPH” subjects during the incongruent condition and of “control MPH” subjects compared to “MA MPH” subjects during the Stroop effect condition. As mentioned earlier, the visual pathways including the occipital cortex may be involved in biasing of selective attention toward the ink color of the presented word stimulus and away from the task-irrelevant and prepotent response of word reading (103). Our results showed that an acute dose of MPH caused a decrease in activation of occipital regions of MA-dependent subjects in comparison to MA-dependent subjects who were treated with placebo. This suggests that MPH intake resulted in a decreased need for occipital activation, while causing a simultaneous improvement in task performance compared to placebo. Further, decreased activation of occipital regions of “MA MPH” subjects compared to “control MPH” subjects suggests an MPH-induced decrease in recruitment of occipital regions for Stroop effect resolution that is specific to active MA-dependent subjects and not healthy controls. Therefore, following an acute dose of MPH, active MA-dependent subjects were able to undergo successful resolution of the Stroop conflict without the need to recruit additional resources within the occipital cortex, while maintaining a comparable level of task performance to healthy controls.

Although there is an established view that the ACC is activated during conditions of information conflict, such as the incongruent condition of the Stroop task, where two streams of information processing compete (34, 36, 37, 40–45, 127), only “control MPH” subjects exhibited increased activation within the ACC and middle cingulate regions during the incongruent and Stroop effect conditions. The increase in activation post- compared to pre-MPH administration of cingulate regions in control subjects may be due to increased regional cerebral blood flow to these regions, as has been previously reported in a positron emission tomography study of healthy subjects treated with an acute dose of MPH (128). Although there was no significant group × drug interaction for RT, “control MPH” subjects exhibited the largest improvement in RT and accuracy post- compared to pre-drug administration across incongruent and Stroop effect conditions, relative to the three other groups. Hence, it is possible that MPH-induced activation of the cingulate cortex may be associated with improved behavioral performance. However, a larger dose of MPH may be required to elicit a similar effect in active MA-dependent subjects.

### Summary of findings

At baseline, all subjects were slower and less accurate during the incongruent compared to the congruent condition. This finding showed that the color-word Stroop task used in the current study probed response inhibition and consequently, cognitive control. However, absence of fMRI activation changes in control subjects corresponding with the Stroop effect condition indicated the task used in the current study could benefit from increased number of blocks/task duration. MA-dependent subjects were slower during congruent and incongruent conditions, possibly reflecting slowed motor responses. During the incongruent condition, MA-dependent subjects showed increased activation of the DLPFC in comparison to control subjects, suggesting their need to recruit frontal neural resources during response inhibition. Although there were no significant between-group differences in Stroop effect RT, MA-dependent subjects exhibited increased activation of the IPL during the Stroop effect condition in comparison to control subjects. It is possible that prior to any treatment, active MA-dependent subjects exhibited a lowered threshold at which incongruent stimuli present sufficient conflict to activate the parietal cortex, thus exhibiting increased activation of the parietal cortex in comparison to control subjects.

Post- minus pre-drug analysis results showed that subjects treated with MPH had lower Stroop effect RT post- compared to pre-drug treatment, relative to those treated with placebo. This suggested that MPH treatment caused an improvement in task performance in both MA-dependent and control subjects. In comparison to “control MPH” and “MA placebo” subjects, “MA MPH” subjects exhibited decreases in fMRI activation of parietal and occipital regions, which were thought to be necessary for Stroop conflict resolution prior to drug treatment. These group differences in fMRI activation suggest that acute MPH treatment in active MA-dependent subjects may normalize the threshold at which incongruent stimuli present sufficient conflict to activate the parietal cortex. Decreases in occipital cortex activation in “MA MPH” subjects suggest that post-MPH treatment, MA-dependent subjects were able to maintain a comparable level of task performance to that achieved pre-drug administration, with a decreased need for recruitment of additional neural resources within the occipital cortex.

### Limitations

There were several limitations to the current study. The sample sizes were small (8 “MA MPH,” 7 “MA placebo,” 8 “control MPH,” and 10 “control placebo” subjects), due to the study having a between-subject design. These sample sizes were smaller than those required by our *a priori* power analysis (129), which was conducted using G*Power v3.1.5 (130). The significance level α was set to 0.05, corrected for multiple comparisons; the power level (1−β) was set to 0.8 and the effect size was estimated at the global maximum activation co-ordinates from a single subject effect size map of a pilot subject scan for the current experiment. The power analysis yielded a sample size of 22 subjects per group. However, only 15 MA-dependent subjects could be recruited, as well as a matching 18 control subjects, who were further divided into two treatment groups. The number of MA-dependent subjects recruited for the MRI studies was limited by the completion of the randomized controlled trial (parent trial) they were recruited from which ended the recruitment process. Moreover, the time difference in the commencement of the parent trial and the MRI studies prevented retrospective recruitment of subjects from the parent trial, due to the commencement of the MPH treatment schedule prior to the MRI studies. Therefore, this study is considered to be underpowered, yielding insightful yet preliminary results, which need to be consolidated by future studies with larger sample sizes.

Another limitation to studying the effects of MA on the brain of active users, and possibly why it is uncommon practice, is the inability to dissociate the effects of long-term use from the potential effects of intoxication and withdrawal (28, 29). However, it is important to investigate the functional effects of MA in active users using neuroimaging as it is this group of drug users who most need to be engaged in clinical trials, in the search for an effective treatment. Furthermore, it is important to report the functional neural correlates of cognitive processes in active MA dependence in order to expand the literature and allow for comparisons to be made with existing literature conducted in short- and long-term abstinent MA-dependent individuals.

Tobacco smoking and cannabis use were more prevalent in the MA-dependent groups. Although it was not possible to recruit active MA-dependent subjects who did not smoke tobacco or use cannabis, future studies should recruit a control group with similar prevalence of tobacco and cannabis use.

Lastly, the use of fMRI to study the effects of an acute challenge of MPH in the current study was not specifically validated in the current study; that is, the possible hemodynamic effects of MPH on fMRI BOLD and blood perfusion were not investigated. However, a previous fMRI study of healthy subjects using a finger tapping task found no changes in task performance or fMRI signals following MPH administration, concluding that MPH did not alter BOLD hemodynamic coupling (131). Consequently, fMRI was validated as a useful tool for studying the neural correlates of cognitive functions under the effect of MPH (131).

## Conclusion

To the best of our knowledge, this is the first fMRI study to investigate the effect of MPH on cognitive control in active MA-dependent subjects in comparison to control subjects. Prior to drug administration, accuracy was lower and RT was longer for all subjects during the incongruent compared to congruent condition, demonstrating the task probed cognitive control. MA-dependent subjects were slower during congruent and incongruent conditions, possibly reflecting poor motor control in comparison to control subjects. During the Stroop effect condition, MA-dependent subjects exhibited increased activation of the inferior parietal cortex compared to control subjects, possibly reflecting a lowered threshold at which incongruent stimuli present sufficient conflict to activate the parietal cortex.

An acute MPH (18 mg) challenge resulted in an improvement in task performance, manifested by a decreased Stroop interference score in all subjects, irrespective of group membership, in comparison to placebo. “MA MPH” subjects showed increased RT and DLPFC activation during the Stroop effect condition post- compared to pre-MPH treatment, suggesting an MPH-facilitated recruitment of the DLPFC for Stroop conflict resolution. In comparison to “control MPH” and “MA placebo” subjects, “MA MPH” subjects exhibited decreases in post- minus pre-drug fMRI activation of parietal and occipital regions, which were thought to be necessary for Stroop conflict resolution prior to drug treatment. Therefore, acute MPH treatment was thought to cause normalization of the threshold at which incongruent stimuli present sufficient conflict to activate the parietal cortex in active MA-dependent subjects, rendering the activation pattern of “MA MPH” subjects indistinguishable from that of untreated healthy control subjects.

The current study provided valuable knowledge about the effects of MPH and placebo on the neural correlates of cognitive control in active MA-dependent and control subjects. Future studies should have a larger sample size and aim to eliminate the confounding effects of other drug use. Furthermore, larger studies of active MA dependence using higher doses of MPH for longer periods are recommended to consolidate the preliminary findings from this study.

## Author Contributions

Reem K. Jan conducted subject recruitment, experimental design, data collection and analysis, the writing of this manuscript, and is the primary author. Joanne C. Lin conducted subject recruitment and data collection and contributed feedback on the written manuscript. Donald G. McLaren provided guidance and intellectual input on data analysis and contributed feedback on the written manuscript. Ian J. Kirk provided guidance and intellectual input on experimental design of the Stroop task. Rob R. Kydd was Reem K. Jan’s secondary PhD supervisor and one of the consultant psychiatrists who conducted screening of MA-dependent subjects and provided intellectual input on all aspects of the research mentioned above. Bruce R. Russell was Reem K. Jan’s primary PhD supervisor, developed the experimental concept, wrote the grants that funded the research and provided intellectual input on all aspects of the research mentioned above.

## Conflict of Interest Statement

The authors declare that the research was conducted in the absence of any commercial or financial relationships that could be construed as a potential conflict of interest.

## Footnotes

^1^http://www.fil.ion.ucl.ac.uk

^2^http://www.nitrc.org/projects/peak_nii
